# Supplementary material for: The Role of Consecutive Plasma Copeptin Levels in the Screening of Delayed Cerebral Ischemia in Poor-Grade Subarachnoid Hemorrhage
Source: Life (Basel). 2021 Mar 25;11(4):274. doi: 10.3390/life11040274 (PMC8066417; doi:10.3390/life11040274)
Supplement: Supplementary file 1 [file life-11-00274-s001.pdf]

life

MDPI

Article

The Role of Consecutive Plasma Copeptin Levels in the Screening of Delayed Cerebral Ischemia in Poor-grade Subarachnoid Hemorrhage

Jong Kook Rhim [1], Dong Hyuk Youn [2], Bong Jun Kim [2], Youngmi Kim [2], Sungeun Kim [3], Heung Cheol Kim [4], and Jin Pyeong Jeon [5,6,*]

1. Department of Neurosurgery, Jeju National University College of Medicine, Jeju 63243, Korea; pedi-neur@daum.net
2. Institute of New Frontier Stroke Research, Hallym University College of Medicine, Chuncheon 24252, Korea; zk61326@naver.com (D.H.Y.); luckykbj@naver.com (B.J.K.); kym8389@hanmail.net (Y.K.)
3. EMS Situation Management Center, Seoul Emergency Operation Center, Seoul Metropolitan Fire & Disaster Headquarters, Seoul 04628, Korea; kimsueu1@hanmail.net
4. Department of Radioilogy, Hallym University College of Medicine, Chuncheon 24252, Korea; khc@hallym.or.kr
5. Genetic and Research Inc., Chuncheon 24252, Korea
6. Department of Neurosurgery, Hallym University College of Medicine, Chuncheon 24252, Korea

* Correspondence: jjs6553@daum.net; Tel: +82-33-240-5171; Fax: +82-33-240-9970

Citation: Rhim, J.K.; Youn, D.H.; Kim, B.J.; Kim, Y.; Kim, S.; Kim, H.C.; Jeon, J.P. The Role of Consecutive Plasma Copeptin Levels in the Screening of Delayed Cerebral Ischemia in Poor-grade Subarachnoid Hemorrhage. *Life* **2021**, *11*, x. https://doi.org/10.3390/life11040274

Academic Editor: Syed Qadri

Received: 02 February 2021
Accepted: 24 March 2021
Published: date

Publisher's Note: MDPI stays neutral with regard to jurisdictional claims in published maps and institutional affiliations.

Copyright: © 2021 by the authors. Submitted for possible open access publication under the terms and conditions of the Creative Commons Attribution (CC BY) license (http://creativecommons.org/licenses/by/4.0/).

Abstract: The prognostic value of copeptin in subarachnoid hemorrhage (SAH) has been reported, but the prognosis was largely affected by the initial clinical severity. Thus, the previous studies are not very useful in predicting delayed cerebral ischemia (DCI) in poor-grade SAH patients. Here, we first investigated the feasibility of predicting DCI in poor-grade SAH based on consecutive measurements of plasma copeptin. We measured copeptin levels of 86 patients on days 1, 3, 5, 7, 9, 11, and 13 using ELISA. The primary outcome was the association between consecutive copeptin levels and DCI development. The secondary outcomes were comparison of copeptin with C-reactive protein (CRP) in predicting DCI. Additionally, we compared the prognostic value of transcranial Doppler ultrasonography (TCD) with copeptin using TCD alone to predict DCI. Increased copeptin (OR = 1.022, 95% CI: 1.008–1.037) and modified Fisher scale IV (OR = 2.841; 95% CI: 0.998–8.084) were closely related to DCI. Consecutive plasma copeptin measurements showed significant differences between DCI and non-DCI groups ($p < 0.001$). Higher CRP and DCI appeared to show a correlation, but it was not statistically significant. Analysis of copeptin changes with TCD appeared to predict DCI better than TCD alone with AUCROC differences of 0.072. Consecutive measurements of plasma copeptin levels facilitate the screening of DCI in poor-grade SAH patients.

Keywords: subarachnoid hemorrhage; copeptin; delayed cerebral ischemia; vasospasm

Supplementary Materials:

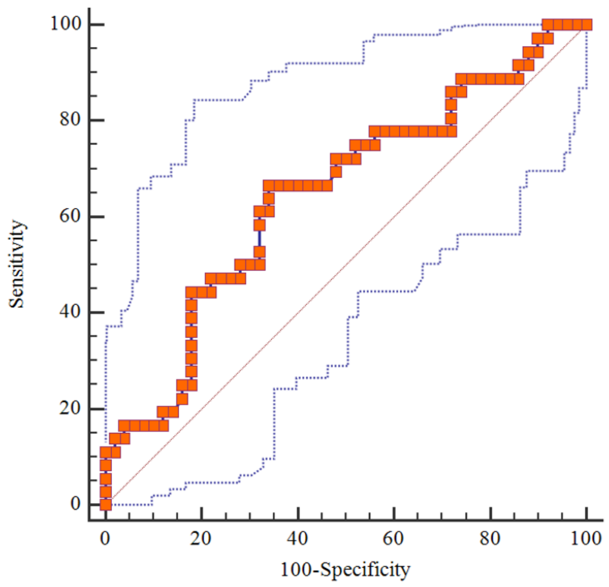

Figure S1. The area under the receiver operator characteristics curve is 0.647. The increase in the rate of plasma copeptin higher than 73% compared with the initial result at admission showed a sensitivity of 66.67% (95% CI: 49.0%–81.4%) and a specificity of 66.00% (95% CI: 51.2%–78.8%). CI= confidence interval. Orange-colored points correspond to criterion values. The blue-dotted lines indicate inclusion of 95% confidence bounds.

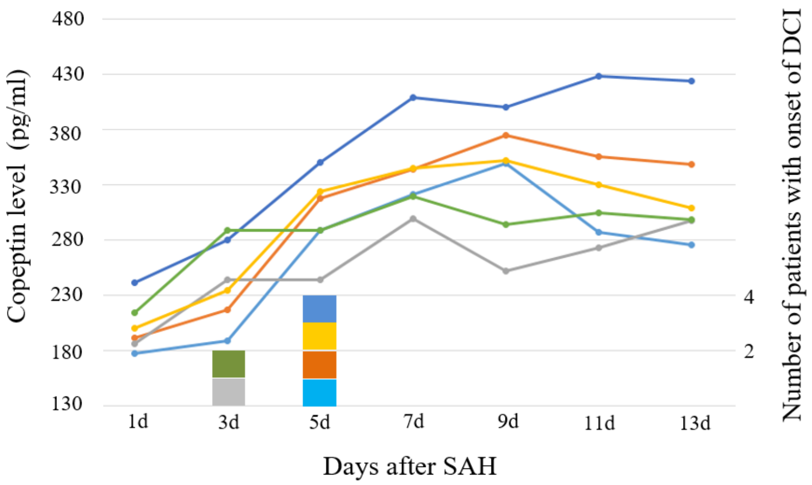

Figure S2. Data of six patients who developed DCI within 5 days after SAH ictus. Consecutive changes in plasma copeptin level and the number of patients with different days of DCI onset are presented. Early increase in copeptin level was more likely to correlate with DCI within 5 days after ictus.

Table S1. Protocol for DCI diagnosis and inter-assessor agreements.

**n = 86**		**Kim's Decision**	
		Non-DCI	DCI
**Rhim's decision**	Non-DCI	48	2
	DCI	3	33

1st Reviewer: Jong Kook Rhim, neurointerventionist with more than 10 years of experience.
2nd Reviewer: Heung Cheol Kim, neuroradiologist with more than 20 years of experience.
3rd Reviewer: Jin Pyeong Jeon, neurointerventionist with more than 6 years of experience.
Each reviewer reviewed medical records and radiological findings independently, followed by a comparison of their DCI diagnosis. Next, Jeon JP reported the process based on inter-assessor reliability with Cohen's kappa index. All disagreements were resolved by Jeon JP. The Cohen's kappa was 0.880, indicating almost perfect agreement.
